# Exploration of the plasma proteomic profile of patients at risk of thromboembolic events

**DOI:** 10.1016/j.rpth.2025.102713

**Published:** 2025-02-28

**Authors:** Eva R. Smit, Iris C. Kreft, Eleonora Camilleri, J. Louise I. Burggraaf-van Delft, Nienke van Rein, Bart J.M. van Vlijmen, Anne-Marije Hulshof, Bas C.T. van Bussel, Frank van Rosmalen, Carmen van der Zwaan, Tom van de Berg, Yvonne Henskens, Hugo ten Cate, Jonathan M. Coutinho, Marieke J.H.A. Kruip, Jeroen J.C. Eikenboom, Arie J. Hoogendijk, Suzanne C. Cannegieter, Maartje van den Biggelaar, in collaboration with BEAT-COVID group and Dutch COVID & Thrombosis Coalition, M. Sesmu Arbous, Bernard M. van den Berg, Suzanne Cannegieter, Christa M. Cobbaert, Anne M. van der Does, Jacques J.M. van Dongen, Jeroen Eikenboom, Mariet C.W. Feltkamp, Annemieke Geluk, Jelle J. Goeman, Martin Giera, Thomas Hankemeier, Mirjam H.M. Heemskerk, Pieter S. Hiemstra, Cornelis H. Hokke, Jacqueline J. Janse, Simon P. Jochems, Simone A. Joosten, Marjolein Kikkert, Lieke Lamont, Judith Manniën, Tom H.M. Ottenhoff, T. Pongracz, Michael R. del Prado, Meta Roestenberg, Anna H.E. Roukens, Hermelijn H. Smits, Eric J. Snijder, Frank J.T. Staal, Leendert A. Trouw, Roula Tsonaka, Aswin Verhoeven, Leo G. Visser, Jutte J.C. de Vries, David J. van Westerloo, Jeanette Wigbers, Henk J. van der Wijk, Robin C. van Wissen, Manfred Wuhrer, Maria Yazdanbakhsh, Mihaela Zlei

**Affiliations:** 1 Department of Molecular Hematology, Sanquin Research, Amsterdam, The Netherlands; 2 Department of Clinical Epidemiology, Leiden University Medical Center, Leiden, The Netherlands; 3 Department of Clinical Pharmacy and Toxicology, Leiden University Medical Center, Leiden, The Netherlands; 4 Department of Internal Medicine, Division of Thrombosis and Hemostasis, Leiden University Medical Center, Leiden, The Netherlands; 5 Cardiovascular Research Institute Maastricht, Maastricht University Medical Center, Maastricht, The Netherlands; 6 Central Diagnostic Laboratory, Maastricht University Medical Centre, Maastricht, The Netherlands; 7 Department of Intensive Care Medicine, Maastricht University Medical Centre, Maastricht, The Netherlands; 8 Department of Internal Medicine, Maastricht University Medical Center, Maastricht, The Netherlands; 9 Department of Neurology, Amsterdam University Medical Centers, University of Amsterdam, Amsterdam, The Netherlands; 10 Department of Hematology, Erasmus MC, Erasmus University Medical Center, Rotterdam, The Netherlands

**Keywords:** biomarkers, mass spectrometry, plasma, proteomics, thrombosis

## Abstract

**Background:**

The elevated health burden of thromboembolic events necessitates development of blood-based risk monitoring tools.

**Objectives:**

We explored the potential of mass spectrometry–based plasma proteomics to provide insights into underlying plasma protein signatures associated with treatment and occurrence of thromboembolic events.

**Methods:**

Utilizing a high-throughput, data-independent acquisition, discovery-based proteomics workflow, we analyzed 434 plasma proteomes from different groups of individuals with elevated risk of thromboembolic events, including individuals I) on vitamin K antagonists (VKAs; *n* = 130), II) with a prior venous thromboembolism (*n* = 10), III) with acute cerebral venous sinus thrombosis (*n* = 10, and IV) with SARS-CoV-2 infection (*n* = 67). Plasma protein levels measured with mass spectrometry were correlated with international normalized ratio and conventional clinical laboratory measurements. Plasma profile differences between different groups were assessed using principal component analysis, moderated *t*-test, and clustering analysis.

**Results:**

Plasma protein levels were in agreement with conventional clinical laboratory parameters, including albumin and fibrinogen. Levels of vitamin K–dependent proteins inversely correlated with international normalized ratio. In the individual studies, we found decreased levels of vitamin K–dependent coagulation proteins in patients on VKAs, alterations in inflammatory signatures among CVST patients and a distinctive signature indicative of SARS-CoV-2 infection. However, no protein signature associated with a thromboembolic event could be identified neither in individual nor combined studies.

**Conclusion:**

Although VKA treatment–specific and disease-specific signatures were captured, our study highlights that the challenges of discovering biomarkers in patients at risk of thromboembolic events lie in the heterogeneity of individual plasma profiles in relation to treatment and etiology.

## Essentials

• Risk stratification for thrombosis requires a better understanding of molecular signatures.
• Unbiased profiling of 420 plasma samples is in strong agreement with clinical laboratory data.
• Identification of vitamin K antagonist treatment and disease-specific proteomic signatures.
• Lack of association to thromboembolic outcome highlights challenges in identifying biomarkers.

## 1 Introduction

Formation and lodging of a thrombus in the vascular bed represents the underlying common pathology of arterial thromboembolism (ATE) and venous thromboembolism (VTE) [1]. Together, ATE and VTE are important determinants of cardiovascular morbidity and mortality, currently the leading cause of death worldwide [2]. Risk assessment scores and models have been developed to predict thromboembolic events in patients at risk of ischemic stroke or patients at risk of (recurrent) VTE [3,4]. These scores are based on general patients’ characteristics, like in the CHA2DS2-VASc score [4] or on a combination of genetic and biochemical markers, such as factor (F)V Leiden in the L-TRRiP score [5], D-dimer levels in the Vienna or HERDOO2 model [6,7]. However, these scores only have a moderate discriminatory ability (C-statistics between 0.6 and 0.7), and therefore, a suboptimal performance in predicting the risk of a future thromboembolic event [3,8]. The recent outbreak of COVID-19 and its association with increased risk of VTE [9] in which current predictive models did not suffice further emphasized the inadequate performance of existing risk prediction scores [10].

The implementation of biomarkers in risk assessment models has been shown to be a powerful tool to refine risk prediction [[11], [12], [13]]. With proteomics techniques capable of measuring multiple proteins simultaneously, numerous studies have used both affinity based as well as mass spectrometry (MS)–based proteomics techniques for biomarker discovery in patients at risk of VTE and ATE [14]. These studies yielded putative biomarkers associated with the risk of developing a thromboembolic event, including inflammatory proteins (eg, serum amyloid [SA] A-1, calcium-binding protein A8) [15], coagulation proteins (eg, vitamin K–dependent protein Z, von Willebrand factor, and tissue factor pathway inhibitor) [16,17], platelet proteins (eg, platelet-derived growth factor subunit B) [18], complement factors (eg, complement factor 5 and complement factor H–related protein 5) [19,20] and apolipoproteins (eg, apolipoprotein C3 and apolipoprotein E) [21]. However, although several of these studies show promising C-statistics, identified biomarkers poorly overlap between studies. This clearly indicates the necessity of additional assessment of proteomic analyses for predicting thromboembolic risks across multiple studies in this field.

Here, we took advantage of recent developments enabling a high-throughput, data-independent acquisition (DIA), MS-based proteomic workflow [[22], [23], [24]] to explore the potential to identify protein signatures in a wide range of study cohorts in the thromboembolic field. To this end, we analyzed plasma profiles from different groups of patients with or at risk of a thromboembolic event, encompassing (I) patients on vitamin K antagonist (VKA) therapy for indication of atrial fibrillation (AF) and VTE, (II) patients with a history of a first VTE, (III) patients with acute cerebral venous sinus thrombosis (CVST), and (V) patients infected with SARS-CoV-2.

## 2 Methods

### 2.1 Study design

**Figure 1:**
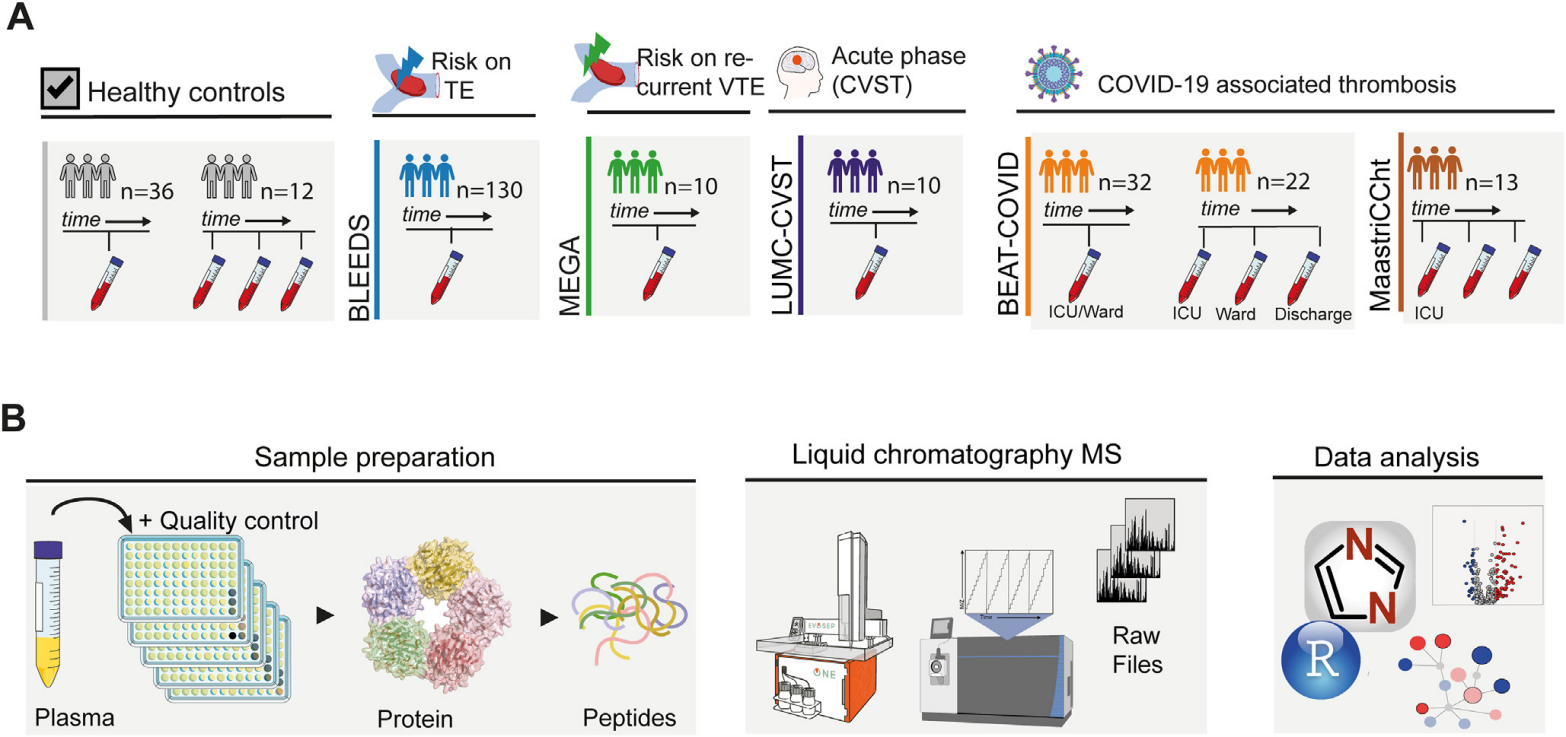
Study design and plasma proteomics. (A) Overview of included cohorts and blood sample collection: healthy controls (gray) both single and serial samples; BLEEDS cohort (blue) patients on vitamin K antagonist for indication atrium fibrillation or previous venous thromboembolism (VTE) or other; MEGA cohort (green) with a VTE history; cerebral venous sinus thrombosis (CVST) cohort (purple); BEAT-COVID (orange) patients with COVID-19 and a risk of VTE included both single and serial samples from ward, intensive care unit (ICU), and after discharge; MaastrICCht cohort (brown) included COVID-19 patients with samples collected during ICU hospitalization. (B) Proteomics workflow starting with plasma sample preparation, followed by liquid chromatography–mass spectrometry (MS) analysis and data analysis. TE, thromboembolism.

To study the potential of unbiased MS-based plasma profiling in the context of thromboembolic events, we combined plasma samples from patients participating in a range of observational cohort studies. These included individuals I) on VKAs for AF or a prior VTE from the Biomarkers in the Leiden Etiology and Epidemiology of bleeding in vitamin K antagonists Drug users study (BLEED study), II) who had a previous first VTE from the Multiple Environmental and Genetic Assessment (MEGA study), III) with an acute CVST from the CVST study, and IV) with a SARS-CoV-2 infection (Biomarker-based Early Anti-inflammatory Therapy for severe COVID-19 [BEAT-COVID study] and Maastricht Intensive Care COVID [MaastrICCht cohort]). Since the patients were included from multiple studies at several hospitals, we also included independent controls participating at the different observational cohort or clinical studies. In addition, we included novel healthy individuals who provided freshly drawn citrated blood plasma to this study (Figure 1A and Supplementary Figure S1). Details on the original study description and plasma isolation is provided in Supplementary Methods.

### 2.2 Patient sample selection

From the BLEED study [25], we selected 56 AF patients without an ischemic stroke, 43 AF patients with an ischemic stroke, 2 AF patients with a VTE, 15 VTE patients without a recurrent VTE, 6 VTE patients with a recurrent VTE and 2 VTE patients with an ischemic stroke. From the MEGA study [26], we included 10 patients with a prior first VTE. Plasma samples from 10 individuals with a CVST [27] were included. From the BEAT-COVID study [28], we selected single time point samples from 27 patients without a VTE and 5 patients with a VTE. Moreover, we selected serial samples from 8 patients without VTE and 14 patients with VTE. From the MaastrICCht cohort [29], we selected serial samples from 8 patients without a VTE and 5 with VTE.

### 2.3 Healthy sample selection

In total, we selected 48 healthy individuals (serving as anonymous controls) from different studies, including 12 individuals from Sanquin, 10 individuals from MEGA, 10 individuals from Prevention of Thrombosis after Knee Arthroscopy, and 12 individuals from BEAT-COVID.

### 2.4 Plasma proteomic workflow

From each sample, plasma was obtained through centrifugation of whole blood. To assess the robustness of our MS-based workflow, we included quality control (QC) samples, which comprised repeated sample injections of a pooled plasma standard from 40 healthy donors. Plasma proteins were denatured, alkylated, reduced, and digested into tryptic peptides as described previously for unbiased analysis [30] (Supplementary Methods). Samples were acidified to a final concentration of 1% (v/v) trifluoroacetic acid (Thermo Fisher Scientific) and diluted with 0.1% formic acid (Biosolve, Netherlands) to a final concentration of 500 ng per 20 μL. For proteomic analysis, peptides were separated using an EvoSep One Liquid Chromatography system (Denmark) coupled to an Orbitrap Fusion Lumos Tribrid mass spectrometer (Thermo Fisher Scientific) operating in DIA mode as described in Supplementary Methods (Figure 1B).

### 2.5 Data analysis

RAW MS files were processed with DIA-NN [22] (version 1.8.1) and searched against the human proteome (reviewed Swiss-Prot Database, 20361 entries, downloaded on April 11, 2022) using a predicted library. Protein inference was based on protein names from FASTA, quantification mode was set to robust liquid chromatography (high accuracy), and match between runs and no shared spectra were enabled without heuristic protein inference strategy. Other settings were set as default. DIA-NN output files were loaded into R (v4.1.1, developed by R Core Team) [31] using tidyverse (v1.3.2, developed by Hadley Wickham et al.) [32] for data processing and visualization purposes. Protein variation was determined by calculating the coefficients of variation (CVs) of non–log_2_-transformed label-free quantification (LFQ) values in proteins quantified in QC samples and separate healthy control studies. For further analysis, QC samples were excluded. Only protein quantifications based on at least 2 different precursors and proteins quantified in at least 50% of all samples in 1 specific cohort were considered for further analysis. Protein levels were log_2_-transformed to ensure optimal performance of linear models assuming Gaussian errors and to reduce the impact of outliers. Missing values were imputed by a normal distribution (width = 0.3; shift = 1.8). Principal component analysis (PCA) was performed on imputed LFQ intensities across all samples color coded by cohort, thrombotic outcome or hospitalization status (only for the COVID-19 cohorts) using prcomp function in the stats package. Statistical significance was determined using moderated *t*-tests from the Limma package, taking individual replicates into account as block function in longitudinal analysis [33]. All *P* values were corrected for multiple testing using the Benjamini–Hochberg method, and *P*-adjusted <.05 and absolute log fold change of >1 was considered statistically significant and relevant.

Spearman correlation was calculated with the Hmisc package [34] for all quantified proteins to international normalized study (INR; BLEED study) and corresponding laboratory data from 2 COVID-19 cohorts. Only correlation values with at least 25% data completeness were kept. Protein dynamics were evaluated using the Pearson correlation coefficients on proteins quantified by means of weighted gene coexpression network analysis using a soft-power of 26 signed network. Modules were defined by dynamic tree cut, and a minimum cluster size of 3 was used [35]. Pathway overrepresentation analyses based on WikiPathways [36] were compared between the clusters using the clusterProfiler package [37] and all identified proteins as background. Only the top-3 most enriched terms for at least 3 proteins with an adjusted *P* value of <.05 were considered relevant. Cluster shapes were determined by *Z*-scored LFQ intensities across all cohorts stratified by thrombotic outcome. Cluster similarity was assessed by calculating the Pearson correlation coefficients of the cluster shapes, selecting only interactions between modules with a Pearson correlation of >0.75. Network visualization was based on cluster similarity, and WikiPathways enrichment terms were visualized using cytoscape [38] with manual protein annotation. All proteins identified, quantified proteins in plasma proteomes across QC, healthy controls and study cohorts as well as correlation with clinical parameters, significant proteins, correlation matrix, and WikiPathways enrichment terms can be found in Supplementary Table S1A–L.

## 3 Results

### 3.1 Stability and reproducibility in plasma proteomics: supporting comparisons

**Figure 2:**
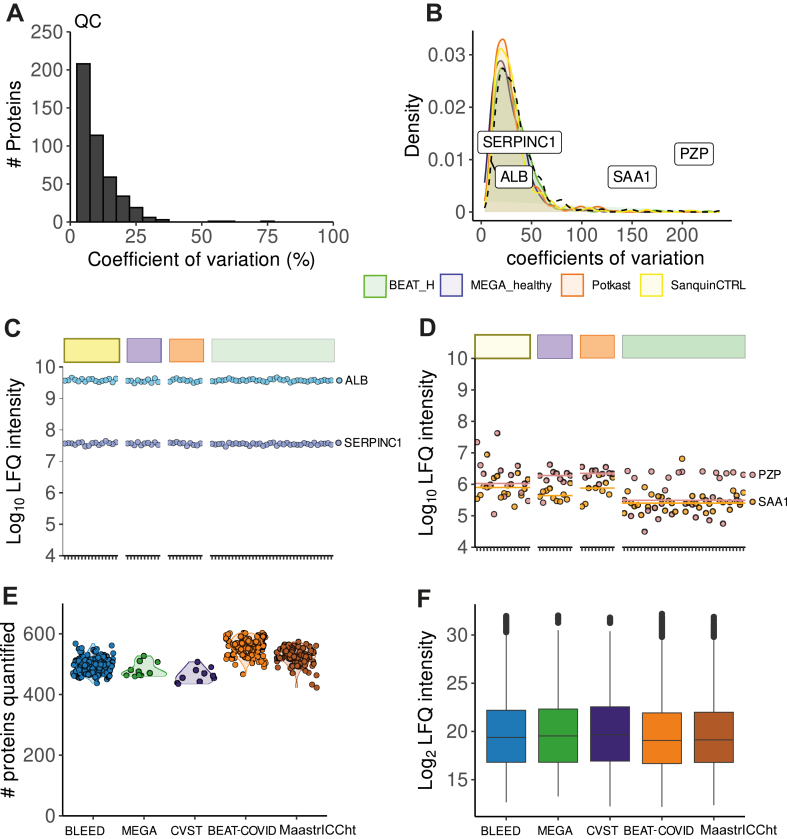
Plasma proteome stability and reproducibility. (A) Histogram representing the coefficients of variation for all proteins quantified in quality control samples (QCs). (B) Density plot of coefficients of variation of quantified proteins per healthy control population. (C) Label-free quantification (LFQ) intensity levels of albumin (ALB, blue) and antithrombin (SERPINC1, purple) across healthy control population (D) LFQ intensity levels of pregnancy zone protein (PZP, pink) and serum amyloid A-1 (SAA1, orange) across healthy control population. (E) Violin plots of quantified proteins across the cohorts. (F) Distributions of LFQ-intensities across the cohorts.

First, we assessed the reproducibility of relative quantification by calculating the CVs for the measured protein levels in the QC samples. A median CV of 7.5% was observed for QC samples, and no bias was detected between different sample preparation plates, reflecting the stability of the workflow (Figure 2A, Supplementary Table S1A). The variation observed in the healthy control study samples were all equally distributed with a median CV of 31.8% (Figure 2B). Among the most stable proteins were albumin (ALB) and antithrombin (SERPINC1) (Figure 2C), whereas pregnancy zone protein and SAA1 protein levels were more variable (Figure 2D). Given the even distribution of protein CVs across all control studies, we opted to combine them into a unified healthy control group for subsequent statistical and global comparative analysis. Across all individual study samples from the different cohorts, we quantified 642 plasma proteins with a median of 492 quantified proteins per cohort (Figure 2E, Supplementary Table S1B, C), with evenly distributed LFQ protein intensities (Figure 2F). Overall, our plasma proteomics workflow showed high reproducibility between technical and biological replicates, and thus, we continued exploring the protein signatures across multiple thromboembolic studies.

### 3.2 Correlation between plasma proteomic measures and laboratory data

**Figure 3:**
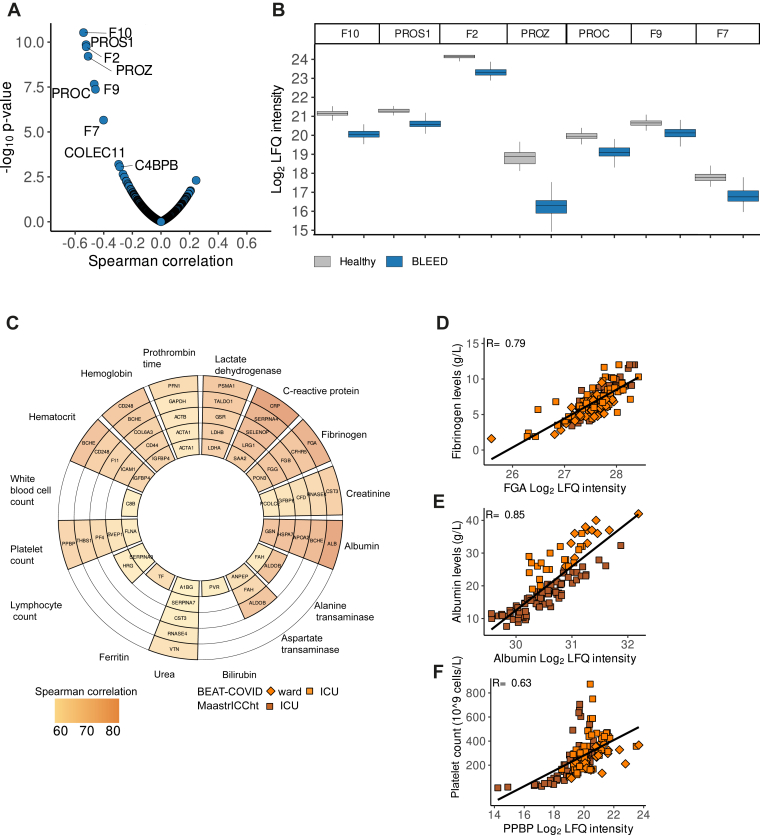
Plasma protein correlation with laboratory data. (A) Correlation analysis of plasma protein levels vs international normalized ratio from BLEED study. The x-axis represents the Spearman correlation coefficient and the y-axis −log_10_*P* values to international normalized ratio. The top 7 absolute highest correlating proteins are labeled by gene name. (B) Boxplots of label-free quantification (LFQ) intensity levels of vitamin k–dependent coagulation proteins: prothrombin (F2), coagulation factor VII (F7), coagulation factor IX (F9), coagulation factor X (F10), vitamin K–dependent protein C (PROC), vitamin K–dependent protein S (PROS1), and vitamin K–dependent protein Z (PROZ) categorized by cohorts (x-axis ticks). (C) Correlation analysis of plasma protein levels vs clinical and laboratory data available from the BEAT-COVID and MaastrICCht cohorts, visualised in a polar plot. Brown gradient depicts strength of correlation represented by Spearman correlation coefficients. (D) Scatter plot shows the correlation between fibrinogen quantified using laboratory measurement (y-axis) and MS acquisition (α chain, FGA, x-axis). Orange, BEAT-COVID, with ward samples (*n* = 12) in squares and intensive care unit (ICU) samples (*n* = 21) in diamonds; brown, MaastrICCht cohort, with only ICU samples (*n* = 77) in diamonds. (E) Scatter plot shows the correlation between albumin quantification by laboratory measurement (y-axis) vs MS acquisition (ALB, x-axis) for BEAT-COVID (ICU samples, *n* = 21; ward samples, *n* = 12) and MaastrICCht (ICU samples, *n* = 79) cohorts. (F) Scatter plot shows the correlation of the platelet count (y-axis) and MS-quantified platelet basic protein (PPBP, x-axis) levels, for BEAT-COVID (ICU samples, *n* = 21; ward samples, *n* = 12) and for MaastrICCht (ICU samples, *n* = 79) cohorts.

Next, we investigated the agreement of our proteomic data with laboratory and clinical parameters that were available from the BLEED study (INR) and from the 2 COVID-19 cohorts (18 laboratory parameters) (Supplementary Methods). We observed an inverse correlation of vitamin K–dependent proteins to INR (Figure 3A, Supplementary Table S1D), including coagulation FX (*r* = −0.54), vitamin K–dependent protein S (PROS1, *r* = −0.52), prothrombin (FII, *r* = −0.52), vitamin K–dependent protein Z (PROZ, *r* = −0.51), vitamin K–dependent protein C (PROC, *r* = −0.47), coagulation FIX (*r* = −0.46) and coagulation FVII (*r* = −0.40), all of which showed notable decrease in protein abundance in samples derived from the BLEED study cohort (Figure 3B). Correlation analysis between proteomic data and the laboratory assays available from the 2 COVID-19 cohorts, resulted in Spearman correlation coefficients of >0.5 for 16 laboratory measurements (Figure 3C, Supplementary Table S1E). Notably, fibrinogen levels quantified through laboratory measurement exhibited a high correlation with fibrinogen alpha chain quantified by MS (*r* = 0.79) (Figure 3C, D), and a similar strong correlation was observed for albumin levels (*r* = 0.85) (Figure 3C–E). Albumin levels were lower in samples obtained from patients at the intensive care unit (ICU), mainly in those from the MaastrICCht cohort, compared with patients at the ward (Figure 3C–E). Furthermore, for platelet counts, we found that the highest correlating proteins were platelet-specific proteins, including proplatelet basic protein (*r* = 0.63), thrombospondin (THBS)-1 (*r* = 0.62) and platelet factor 4 (*r* = 0.61) (Figure 3C–F).

### 3.3 Global plasma proteome variation driven by inflammatory proteins

**Figure 4:**
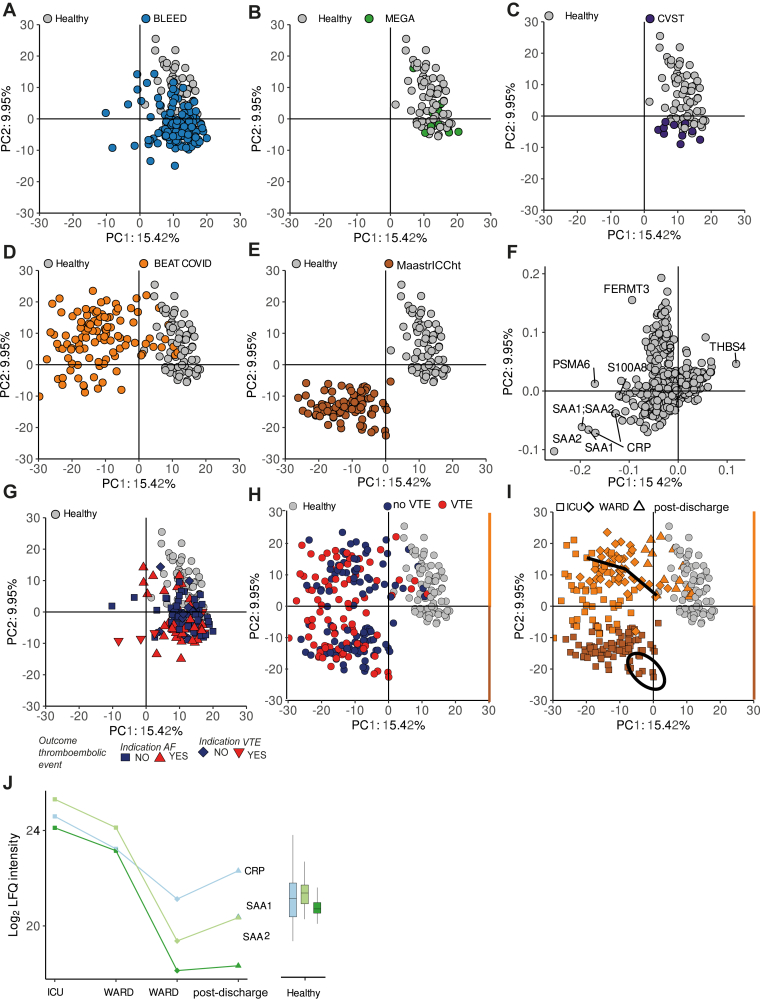
Plasma proteomics identifies cohort-specific and individual-specific signatures. Principal component analysis (PCA) based on imputed label-free quantification (LFQ) values from 642 quantified proteins annotated by cohort for (A) BLEEDS (blue) vs healthy controls (gray); (B) MEGA (green) vs healthy controls (gray); (C) cerebral venous sinus thrombosis (CVST; purple) vs healthy controls (gray); (D) BEAT-COVID (orange) vs healthy controls (gray); and (E) MaastrICCht cohort (brown) vs healthy controls (gray). (F) Loadings protein plot of PCA that highlights inflammatory-related proteins as key determinants of variance, for the most important principal components (PC1 and PC2). (G) BLEED study cohort annotated based on indication, including atrial fibrillation (AF) indication without thrombotic outcome (square) and with future thrombotic outcome (triangle point up) as well as venous thromboembolism (VTE) indication without thrombotic outcome (circle) and with future thrombotic outcome (triangle point down). (H) COVID-19 patients, including the BEAT-COVID and MaastrICCht cohorts, representing patients with future thrombotic outcome (red) and patients without thrombotic outcome (blue). (I) COVID-19 patients, including the BEAT-COVID and MaastrICCht cohorts, annotated by hospitalization status at time of sampling. For BEAT-COVID, samples were collected while patients were on the intensive care unit (ICU; orange squares), ward (orange diamonds) and postdischarge (orange triangles pointed upward). For MaastrICCht cohort, samples were collected during ICU (brown squares). (J) Changes in the protein levels of C-reactive protein (CRP, light blue), serum amyloid A-1 protein (SAA1, light green), and serum amyloid A-2 protein (SAA2, dark green) depicted for patient X from the BEAT-COVID cohort for whom samples were collected during the stay at the ICU, ward (2 time points), as well as postdischarge. The right panel shows the LFQ distribution for each protein over all healthy control samples.

To explore the plasma proteomes of all patients that participated in the various cohort studies in relation to healthy controls and the individual cohorts, we performed PCA. This analysis showed that patients in the BLEED, MEGA, and CVST studies were in close proximity to the healthy control population (Figure 4A–C), while the 2 COVID-19 cohorts clearly separated along the first principal components (PC1 and PC2) (Figure 4D, E). As expected, inflammatory-related proteins including C-reactive protein (CRP), SAA1, S100 calcium-binding protein (S100) A8, and S100A9 were the largest drivers of the variation observed in the PCA (Figure 4F). The global proteome did not distinguish between patients on VKA who did or did not develop a thromboembolic event (Figure 4G) and COVID-19 patients who did or did not develop a VTE (Figure 4H). Nevertheless, for the COVID-19 patients, we did find clustering based on hospitalization status (ICU, ward, or discharge) (Figure 4I), showing that the main drivers of plasma proteomes (CRP, SAA1, and SAA2) can effectively function as clinical classifiers for disease severity (Figure 4J).

### 3.4 Specific protein signatures associated with VKA treatment, CVST, and COVID-19

**Figure 5:**
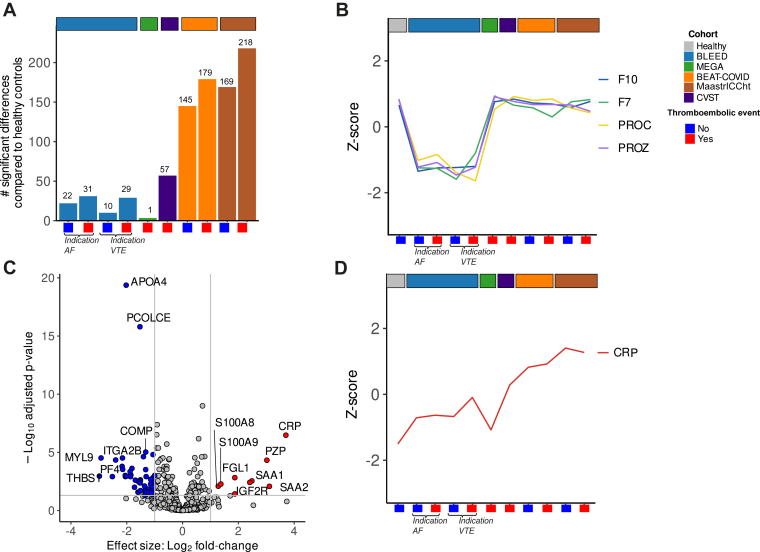
Proteomic differences between healthy controls and multicentre cohorts (A) Number of statistically significant altered proteins comparing of cohorts with (red) and without (blue) future thrombotic events across thrombosis cohorts vs healthy controls (Benjamini–Hochberg adjusted *P* < .05) and effect size (|logFC| > 1). (B) Boxplot of LFQ intensities of vitamin K–dependent protein Z (PROZ), vitamin K–dependent protein C (PROC), factor 10 (F10), and factor 7 (F7) plotted over subgroups and cohorts. (C) Volcano plot of statistical significance against log2-fold change between patients with cerebral venous sinus thrombosis (CVST) and healthy controls. Proteins with significant (*P*_adj_ < .05) differences in abundance (nonsignificant proteins colored gray) with a logfold change of >1.0 (red) and <1.0 (blue). For all analyses, *P* values were adjusted for multiple hypothesis testing using the Benjamini–Hochberg method. (D) Median *Z*-scored LFQ intensities of C-reactive protein (CRP) stratified per patient subgroup. AF, atrial fibrillation; VTE. venous thromboembolism.

To investigate plasma protein alterations associated with the development of thrombosis, we compared patients from each study, stratified for the occurrence of thromboembolic event, to the healthy controls using statistical analysis (Supplementary Table S1F). Our findings revealed the least number of significantly different proteins for the MEGA study, followed by the BLEED study and CVST. As expected, COVID-19 cohorts had the most significant differences compared with healthy controls (Figure 5A). In the BLEED study, the most significantly different proteins were VKA-dependent proteins F10, PROZ (in all subgroups compared with healthy controls) and PROC and F7 (in the patients with VTE as indication compared with healthy controls) (Supplementary Figure S2, Figure 5B, Supplementary Table S1G). In patients with CVST, in which the blood sampling was performed in the acute phase after the CVST, the abundance of inflammatory-related proteins (eg, CRP, SAA1, SAA2, S100A8, S100A9, fibrinogen-like protein 1) was increased compared with healthy controls, along with a decrease in abundance for platelet-related proteins (eg, platelet factor 4, proplatelet basic protein, THBS-1, filamin A integrin subunit α2b) and apolipoprotein A4 (Figure 5C). Within both COVID-19 cohorts, independent of the occurrence of thromboembolic outcome, inflammatory-related proteins (eg, leucine-rich α2-glycoprotein 1, S100A8, S100A9, CRP, and SAA1), von Willebrand factor (VWF) and neutrophil-related proteins (eg lactotransferrin, myeloperoxidase, and neutrophil defensin 1) were increased in abundance compared with healthy controls (Supplementary Figure S3, Supplementary Table S1G). Interestingly, CRP levels were increased in 8 of the 10 subgroups compared with healthy controls, irrespective of the thromboembolic outcome (Figure 5D, Supplementary Table S1G). Given the higher number of significantly altered proteins in the patients with a thromboembolic event compared with healthy controls (for all studies, Figure 5A), we next compared the plasma proteome profiles of thromboembolic outcome subgroups within each cohort separately. In the BLEED study, this revealed no significant altered proteins when comparing the BLEED study patients with AF with and without an ischemic stroke, and only 1 alteration (immunoglobulin κ variable 1D-33) when comparing BLEEDS patients treated for a prior VTE with and without recurrent VTE (Supplementary Table S1H). In the BEAT-COVID study, we did observe significant alterations in 21 proteins (eg, matrix metalloproteinase-9, neutrophil gelatinase-associated lipocalin, and osteopontin, v-set and immunoglobulin domain-containing protein 4, and lactotransferrin) between the patients who did or did not develop VTE (Supplementary Figure S4A, B, Supplementary Table S1G). However, none of these 21 proteins were significantly different between the VTE and non-VTE group in the MaastrICCht cohort (Supplementary Figure S4B, Supplementary Table S1H) and did not show similar trends in protein levels between the 2 groups either (Supplementary Table S1I).

### 3.5 Unbiased clustering analysis demonstrated protein dynamics across multicenter studies

**Figure 6:**
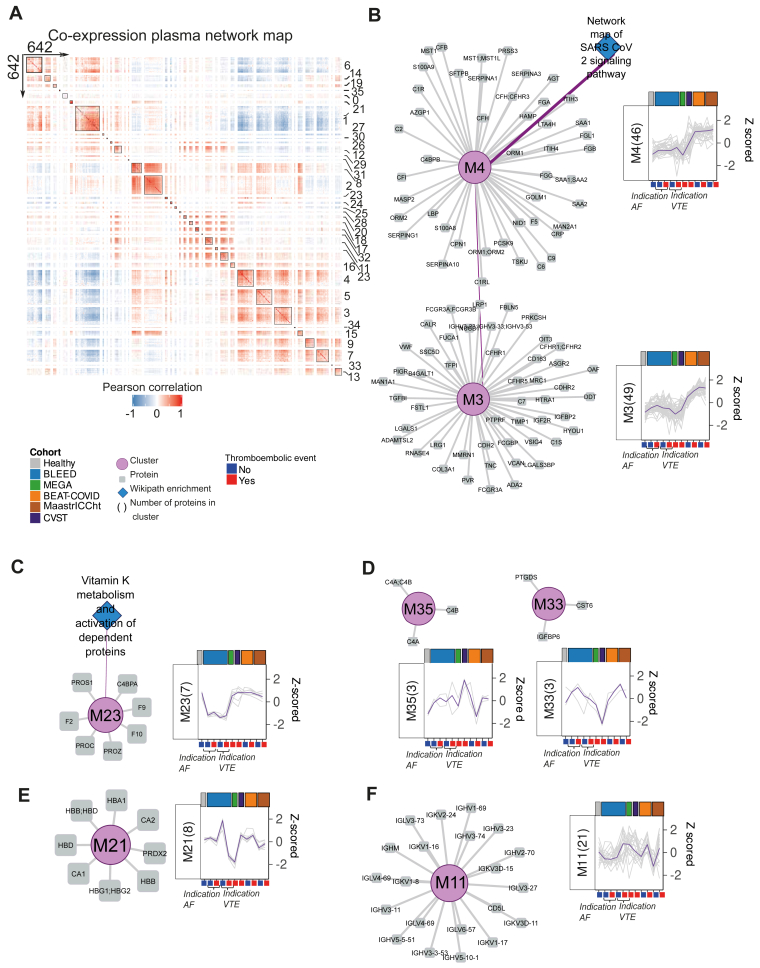
Protein signatures and dynamics related to underlying etiology. (A) Heatmap of Pearson correlation coefficient of the pairwise comparison of 642 quantified proteins in this study. Column and row splits are based on weighted gene coexpression network analysis–defined functional modules, indicated with numbers. Color gradient indicated Pearson correlation coefficients (blue, −1; white, 0; red, 1). (B) Network representation of clusters 3 and 4; (C) cluster 23 with (D) cluster 35; (E) cluster 21; (F) cluster 11, from heatmap with corresponding enriched Wikipathway terms represented with blue diamond. Cluster likeness (Pearson correlation coefficient ≥ 0.75 or a shared Wikipathway term) is indicated as connecting purple edges. Proteins are represented as gray squares. Line graphs show *Z*-scored protein intensities for each cluster (individual proteins, gray lines; median of cluster, purple line). AF, atrial fibrillation; VTE. venous thromboembolism.

In search of cohort-specific and thromboembolic-specific molecular signatures, we took advantage of the observed contrasts across the cohorts to cluster the dynamics of all 642 quantified proteins in the whole data set using weighted gene coexpression network analysis. This resulted in 35 clusters varying in size from 3 to 70 proteins that were constructed in a global correlation network map (Figure 6A, Supplementary Table S1J), 8 of which were enriched for Wikipathways (Supplementary Table S1K).

Next, we visualized the protein trends over all cohorts grouped by thrombotic outcome for each cluster (Supplementary Figure S5). In line with global variance and statistical analysis, global correlation analysis revealed a clear COVID-19 signature. Proteins in cluster M4, which enriched for network map of SARS-CoV-2 signaling pathway, were associated with both COVID-19 cohorts. In this study, we observed altered levels in inflammatory-related proteins (eg, SAA1, CRP, and SAA2) (Figure 6B). Cluster M3 was highly correlated to cluster M4 as indicated by a high Pearson correlation coefficient (*r* = 0.92; Supplementary Table S1L) between the 2 clusters, and contains proteins with a similar alteration in COVID-19 cohorts (eg, VWF, leucine-rich α2-glycoprotein 1, galectin-1, and polymeric immunoglobulin receptor). Furthermore, consistent with Figure 2A, proteins in cluster M23, including vitamin K–dependent coagulation proteins PROS1, F9, F10, PROZ, PROC, F2, and complement 4 binding protein (C4BP) A, exhibited decreased levels across all BLEED study subgroups (Figure 6C). Two distinct clusters specific to CVST were identified, with cluster M33 including cystatin E/M9, insulin-like growth factor binding protein 6, and prostaglandin D2 synthase showing decreased protein levels and cluster M35 including complement C4A, C4B, and total C4 exhibiting increased levels (Figure 6D). Cluster M21 contained hemoglobin subunits (HBA1, HBB, HBD, HBG1, and HBG2), carbonic anhydrases 1 and 2 and peroxiredoxin 2 that were decreased in abundance in the MEGA study and in the subgroup of BLEED study patients with recurrent VTE (Figure 6E). Furthermore, in search for a specific cluster with protein dynamics associated with thromboembolic outcome, we noted that cluster M11, including cluster of differentiation (CD) 5 like, immunoglobulin heavy constant μ and 19 variable regions of immunoglobulin κ and λ chains, displayed slightly elevated levels in patients who developed a VTE compared with patients who developed an ischemic stroke, patients without a thromboembolic outcome, and healthy controls (Figure 6F).

## 4 Discussion

In search of biomarkers to improve diagnostics and risk prediction in thromboembolic events, a range of proteomic approaches, including affinity- and MS-based proteomics, have been used [14]. This has resulted in a wide variety of candidate biomarkers, most of which are poorly reproducible between studies and may therefore be cohort specific. In this study, we set out to examine if discovery-based proteomics can provide insight into the association of protein abundance to thromboembolic events from multiple studies in the thromboembolic field. To this end, we analyzed 420 plasma samples from 219 participants and 14 QC samples using state-of-the art shotgun proteomic technology enhanced with DIA featuring high sensitivity. While our discovery-driven approach detected a large array of proteins with high overlap compared with the described studies [14], we did not observe significant alterations of many of the putatively reported biomarkers based on thromboembolic outcome.

The high correlation of protein levels measured with MS to levels measured with conventional laboratory testing, as demonstrated for, eg, albumin and fibrinogen, highlights the potential of unbiased proteomics in the field of thrombosis and hemostasis. For all vitamin K–dependent coagulation proteins that contain a γ-carboxyglutamate domain [39], we found that levels were reduced in the VKA-treated cohort (BLEEDS) compared with healthy controls. This is not unexpected since the working mechanism of VKA is based on inhibition of vitamin K epoxide reductase, a cofactor required for carboxylase-mediated γ-glutamyl carboxylation of glutamic acid residues in the γ-carboxyglutamate domains [39,40]. Our data indicate that VKA treatment not only decreases the function but also the abundance levels of these proteins in plasma. Interestingly, we observed heterogeneity in the relative reduction of the different VKA-dependent coagulation proteins, with FX correlating strongest to INR, while PROZ was most substantially reduced. Whether this heterogeneity results from differences in the number of affected glutamate residues or on the impact thereof on protein folding, secretion, or half-life/clearance due to structural instability [41] remains a topic for future studies. To further address this topic requires longitudinal monitoring of patients before, during and after VKA treatment. Furthermore, additional DDA-based approaches beyond discovery-based proteomics approaches can potentially enable protein-specific personalized assessment of the VKA-treatment response, including identification and quantification of the negatively charged γ-carboxylated peptides. Alternatively, native MS-based approaches might shed light on the extent of intact proteoforms during VKA treatment.

Both in the comparison of all cohorts as well as in comparison of individual cohorts with healthy controls, inflammatory proteins constituted the largest drivers of the plasma proteome variation. This was to be expected for the COVID-19 patients and CVST groups, as an inflammatory response is associated with viral infection [42] and acute thrombosis [43,44]. In line with literature, we found that for CVST patients the levels of S100A8 [15,16], S100A9 [16], and SAA1 [15], which have been associated with thromboembolic outcome, were increased in abundance compared with healthy controls. Moreover, we also observed several other inflammatory proteins (eg, CRP, SAA2, and fibrinogen-like protein 1) that showed increased levels, providing further support of the inflammatory response during acute thromboembolic events. Interestingly, CRP plasma levels were significantly higher in 8 of the 10 study groups, when compared with healthy controls. The observations that CRP and other inflammatory proteins levels are increased align with the well described link between inflammation and thrombosis [43], but do not assess causation. To establish this cause-and-effect relationship would require larger and more homogenous matched prethromboembolic and postthromboembolic cohorts through population-based studies, which would enable controlling for interfering factors, including treatment, age, and sex.

The recent SARS-CoV-2 outbreak and its association with increased risk of thrombosis [45] emphasized the inadequate performance of existing risk prediction scores for future thromboembolic events. In line with plasma and serum protein profiling studies on the changes in plasma or serum proteomes associated with a SARS-CoV-2 infection [22,24,46,47], we found disease-specific signatures indicative of SARS-CoV-2 infection in COVID-19 patients. A limited number of studies have been performed on the increased risk of thromboembolic outcomes in these patients [48,49]. Elevated levels of VWF have been linked to the increased frequency of thromboembolism events in COVID-19 patients [42]. Our study indeed observed significant alterations in VWF levels in both COVID-19 cohorts, yet no significant differences were found between COVID-19 patients who did or did not develop VTE. Besides VWF, acute inflammation characterized by elevated acute-phase proteins and cytokines has been suggested to play a role in the development of thrombosis in COVID-19 patients [42,50]. Our results in the BEAT-COVID study may support this hypothesis with alterations in platelet-related proteins (THBS-4 and matrix metalloproteinase 9), endothelial-related proteins (endosialin [CD248]) and immune-related proteins (mannose receptor c-type1 and lipocalin 2). However, these alterations were not observed in the MaastrICCht cohort. The plasma samples from MaastrICCht cohort were obtained from patients at the ICU, whereas in the BEAT-COVID study, plasma samples were obtained from patients at the ICU, ward, and postdischarge. The differences in hospitalization status and severity could potentially explain these differences. In fact, previous studies also showed severity of COVID-19 is associated with increased levels of inflammatory proteins (interleukin 6) [51], coagulation proteins (VWF [52] and fibrinogen [53]), but a specific link with development of thromboembolic outcome could not be found [49].

The inclusion of plasma samples derived from multiple studies offered an opportunity for a systematic and broad exploration of plasma proteome dynamics in the thromboembolic field using co-expression analysis. This approach has been demonstrated to link intricate processes within the plasma proteome and associated proteins to common functions and clinical risk measures [47,54,55]. We reiterate the power of this approach by highlighting a single cluster of vitamin K–dependent proteins (F2, F9, F10, PROS1, PROC, and PROZ), also including the PROS1-interacting protein C4BP. Interestingly, this cluster does not contain the C4BP β chain (C4BPB) that interacts with PROS1 [56]. This is in line with the observation that the ratio of the C4BPA:C4BPB decreases during inflammation [57] and in support of a direct relation between the coagulation and complement systems [56]. One additional observation is that the only cluster that shows a correlation (albeit weak) with VTE-prone patient subgroups is cluster M11, including the highly correlating proteins immunoglobulin heavy constant μ and CD5L [58,59]. Interestingly, Obermayer et al. [60] have recently suggested that immunoglobulin M antibodies directed against oxidation-specific epitopes are modulators of coagulation and may be protective against thrombosis.

Our study has several limitations that should be taken into consideration when interpreting the findings. As this was a study in which we included groups of patients from different studies, some of the patient subgroups were small, limiting the power to detect significant differences. The unclarity in optimal cutoff level that defines increased risk for many biomarkers [49], combined with the stringent criteria to determine significant relevance of protein changes used in this study, may result in the oversight of minor alterations in proteins that could be associated with the risk of thromboembolic events. Additional study-specific limitations included the lack of consistency in thrombosis diagnostic workup in the 2 cohorts of COVID-19 patients that may result in missed thromboembolic events [61], as well as the impact of additional pharmacological treatment and other comorbidities at the time of sampling, for example, immunosuppressive treatment. Moreover, the time between the time point of sample collection and the time point of the (first) thromboembolic event was variable between studies, which added additional variability. Lastly, the variations in sample preparation method across the multiple cohorts may have induced some bias as illustrated by cluster M21, which contained previously described erythrocyte contaminants [62]. Although these limitations prohibit us from making strong conclusions, this study is the first to demonstrate the value of high-throughput DIA workflows in discovery-based thrombosis research, revealing proteome profiles that are affected by treatment and underlying conditions, such as the impact of VKAs and the COVID-19. In addition, it emphasizes the need for large-scale, collaborative studies with standardized protocols and rigorous QC measures to minimize the effect of sample-handling procedures and optimize the potential to identify predictive biomarkers for thromboembolic events [62].

## Appendices

BEAT-COVID group members: M. Sesmu Arbous (Leiden, The Netherlands), Bernard M. van den Berg (Leiden, The Netherlands), Suzanne Cannegieter (Leiden, The Netherlands), Christa M. Cobbaert (Leiden, The Netherlands), Anne M. van der Does (Leiden, The Netherlands), Jacques J. M. van Dongen (Leiden, The Netherlands), Jeroen Eikenboom (Leiden, The Netherlands), Mariet C. W. Feltkamp (Leiden, The Netherlands), Annemieke Geluk (Leiden, The Netherlands), Jelle J. Goeman (Leiden, The Netherlands), Martin Giera (Leiden, The Netherlands), Thomas Hankemeier (Leiden, The Netherlands), Mirjam H. M. Heemskerk (Leiden, The Netherlands), Pieter S. Hiemstra (Leiden, The Netherlands), Cornelis H. Hokke (Leiden, The Netherlands), Jacqueline J. Janse (Leiden, The Netherlands), Simon P. Jochems (Leiden, The Netherlands), Simone A. Joosten (Leiden, The Netherlands), Marjolein Kikkert (Leiden, The Netherlands), Lieke Lamont (Leiden, The Netherlands), Judith Manniën (Leiden, The Netherlands), Tom H. M. Ottenhoff (Leiden, The Netherlands), T. Pongracz (Leiden, The Netherlands), Michael R. del Prado (Leiden, The Netherlands), Meta Roestenberg (Leiden, The Netherlands), Anna H. E. Roukens (Leiden, The Netherlands; Principal investigator and lead author [A. H. E. Roukens], email: A.H.E.Roukens@lumc.nl), Hermelijn H. Smits (Leiden, The Netherlands), Eric J. Snijder (Leiden, The Netherlands), Frank J. T. Staal (Leiden, The Netherlands), Leendert A. Trouw (Leiden, The Netherlands), Roula Tsonaka (Leiden, The Netherlands), Aswin Verhoeven (Leiden, The Netherlands), Leo G. Visser (Leiden, The Netherlands), Jutte J.C. de Vries (Leiden, The Netherlands), David J. van Westerloo (Leiden, The Netherlands), Jeanette Wigbers (Leiden, The Netherlands), Henk J. van der Wijk (Leiden, The Netherlands), Robin C. van Wissen (Leiden, The Netherlands), Manfred Wuhrer (Leiden, The Netherlands), Maria Yazdanbakhsh (Leiden, The Netherlands), Mihaela Zlei (Leiden, The Netherlands)
