# Supplementary material for: Exploration of the plasma proteomic profile of patients at risk of thromboembolic events
Source: Res Pract Thromb Haemost. 2025 Feb 28;9(2):102713. doi: 10.1016/j.rpth.2025.102713 (PMC11986537; doi:10.1016/j.rpth.2025.102713)
Supplement: Supplementary Figures [file mmc1.docx]

**Supplemental Figures**


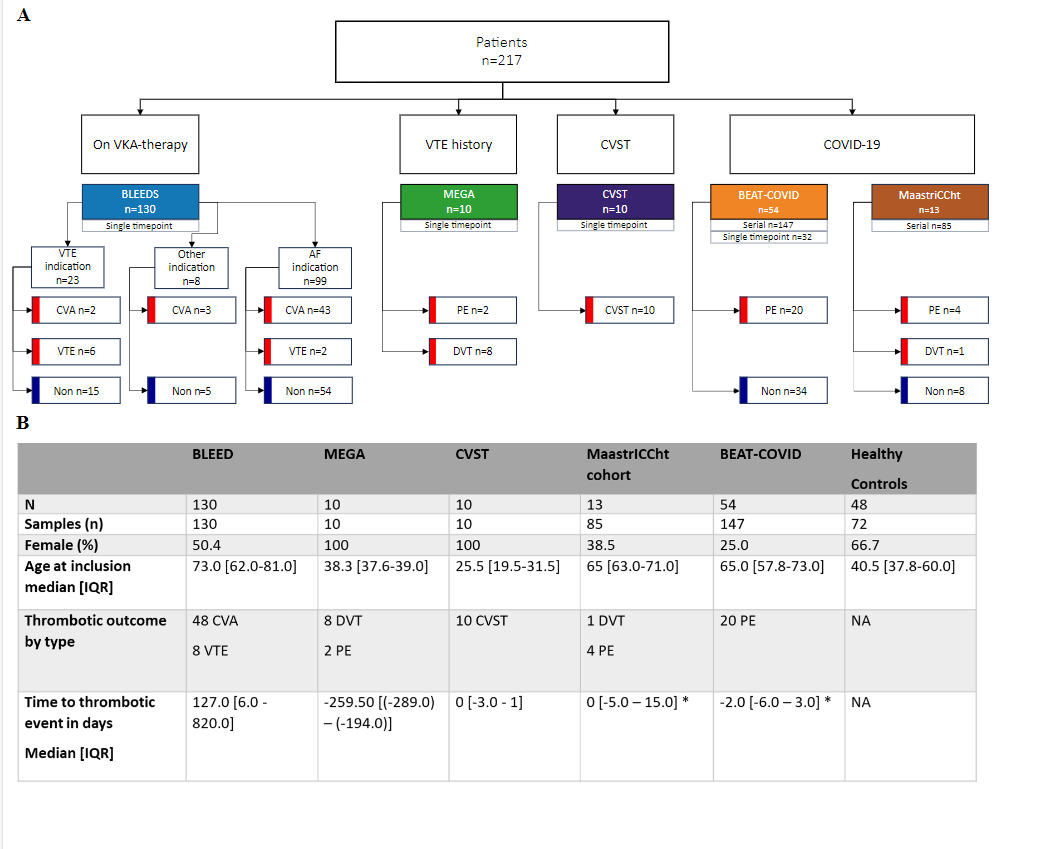


**Supplemental Figure S1**: A: Distribution of the patients included in this study separated across the cohorts. For each cohort the distribution of thrombosis etiology is annotated. Red vertical bar represents thrombosis groups, and blue non-thrombosis group. B: Patient characteristics. * distance calculated based on the first sample for patients with longitudinal sampling. CVA=CerebroVascular Accident; PE=Pulmonary embolism; DVT=deep vein thrombosis; CVST=cerebral venous sinus thrombosis; VTE=venous thrombosis embolism; TIA=Transient Ischaemic Attack. NA: Not Applicable, VTE: Venous Thromboembolism, BLEED: the Biomarkers in the Leiden Etiology and Epidemiology of bleeding in vitamin K antagonists Drug users; MEGA: the Multiple Environmental and Genetic Assessment; BEAT-COVID: Biomarker-based Early Anti-inflammatory Therapy for severe COVID-19; MaastrICCht: Maastricht Intensive Care COVID.


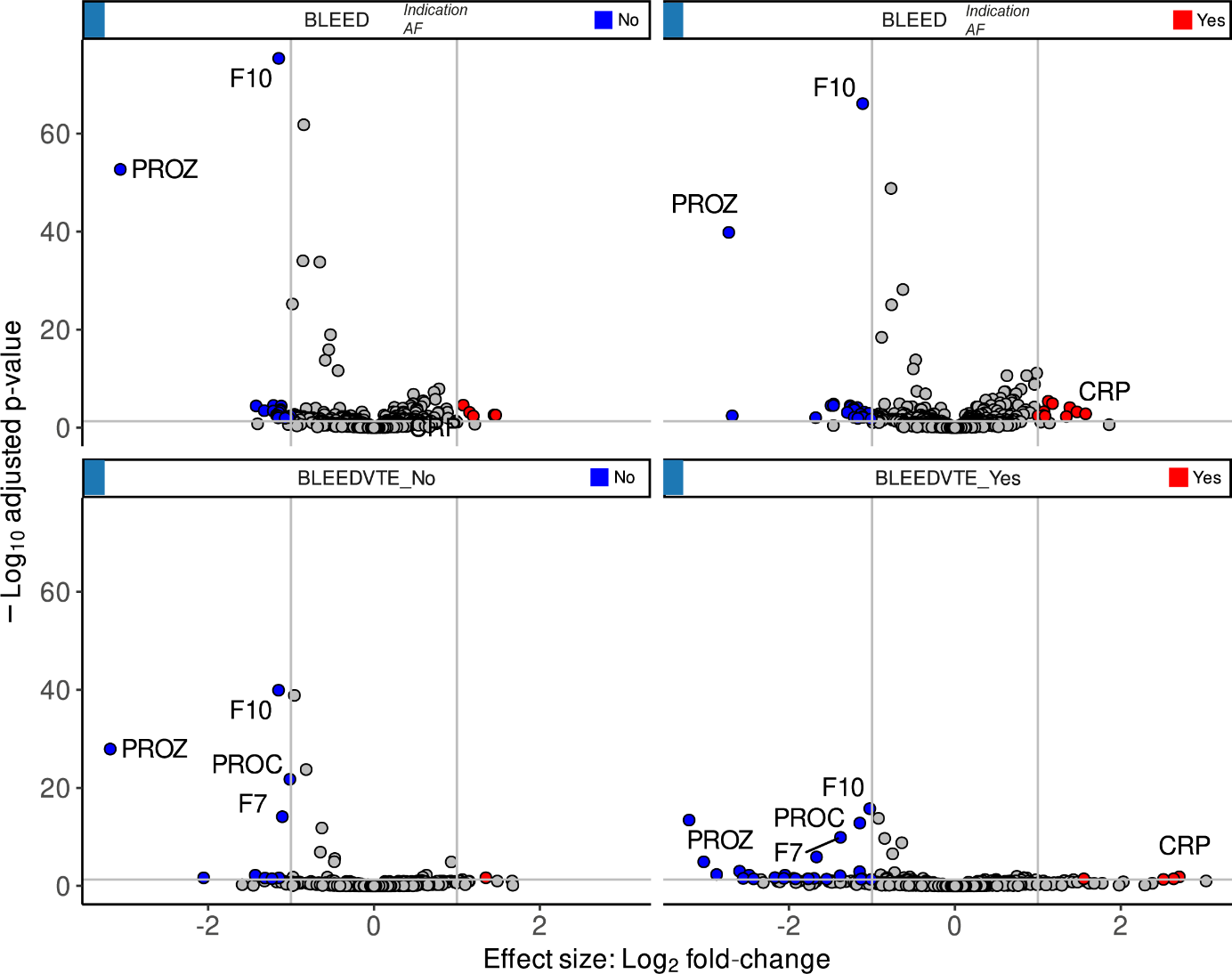


**Supplemental Figure S2**: Volcano plots displaying differentially expressed plasma proteins in the BLEEDS when comparing patients subgroups to healthy controls. Proteins with significant (Padj < 0.05) differences in abundance (nonsignificant proteins colored grey) with a log fold change >1.0 (in red) and <1.0 (colored in blue). For all analyses, P values were adjusted for multiple hypothesis testing using the Benjamini and Hochberg method.


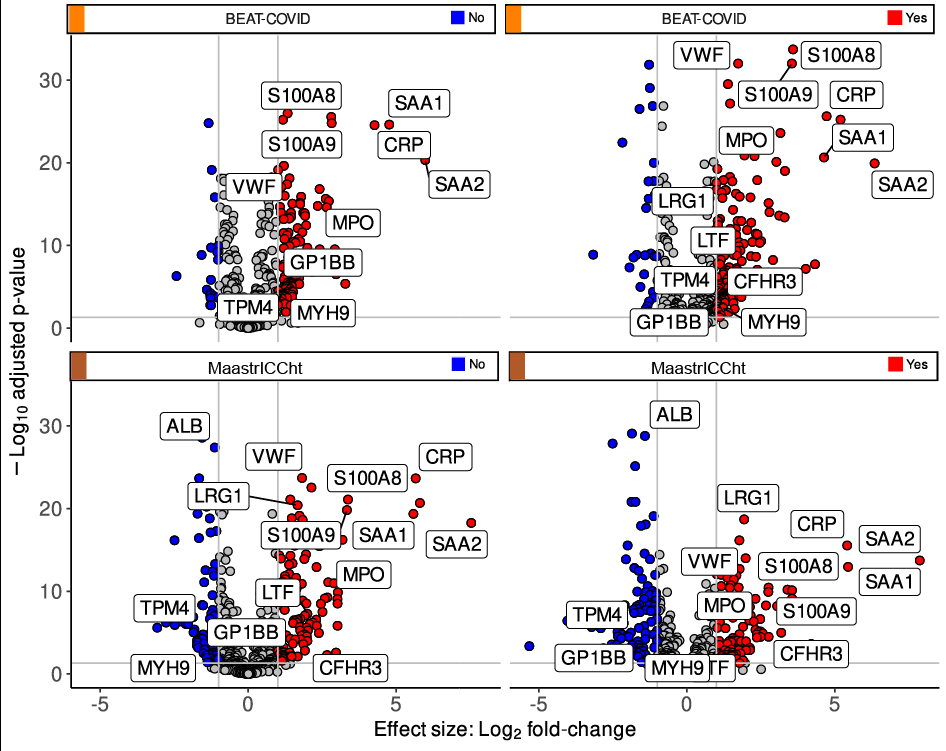


**Supplemental Figure S3:** Volcano plots displaying differentially expressed plasma proteins in the discovery study when comparing COVID-19 cohorts (BEAT-COVID and MaastrICCht) with and without thromboembolic outcome compared to healthy controls. Proteins with significant (Padj < 0.05) differences in abundance (nonsignificant proteins colored grey) with a log fold change >1.0 (in red) and <1.0 (colored in blue). For all analyses, P values were adjusted for multiple hypothesis testing using the Benjamini and Hochberg method.


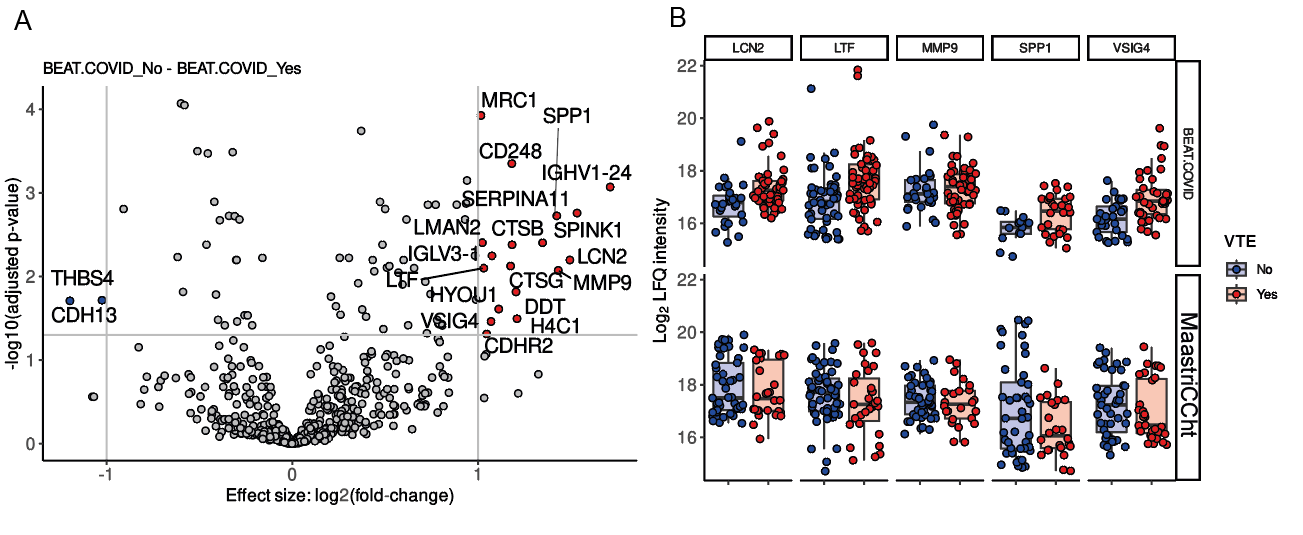


**Supplemental Figure S4:** (A) Volcano plot of statistical significance against log2-fold change between COVID-19 patients between COVID-19 patients with and without a thromboembolic event. Proteins with significant (Padj < 0.05) differences in abundance (nonsignificant proteins colored grey) with a log fold change >1.0 (in red) and <1.0 (colored in blue). For all analyses, P values were adjusted for multiple hypothesis testing using the Benjamini and Hochberg method. (B) boxplot of the differently expressed proteins in A, plotted for COVID-19 cohorts and stratified based on thromboembolic outcome.


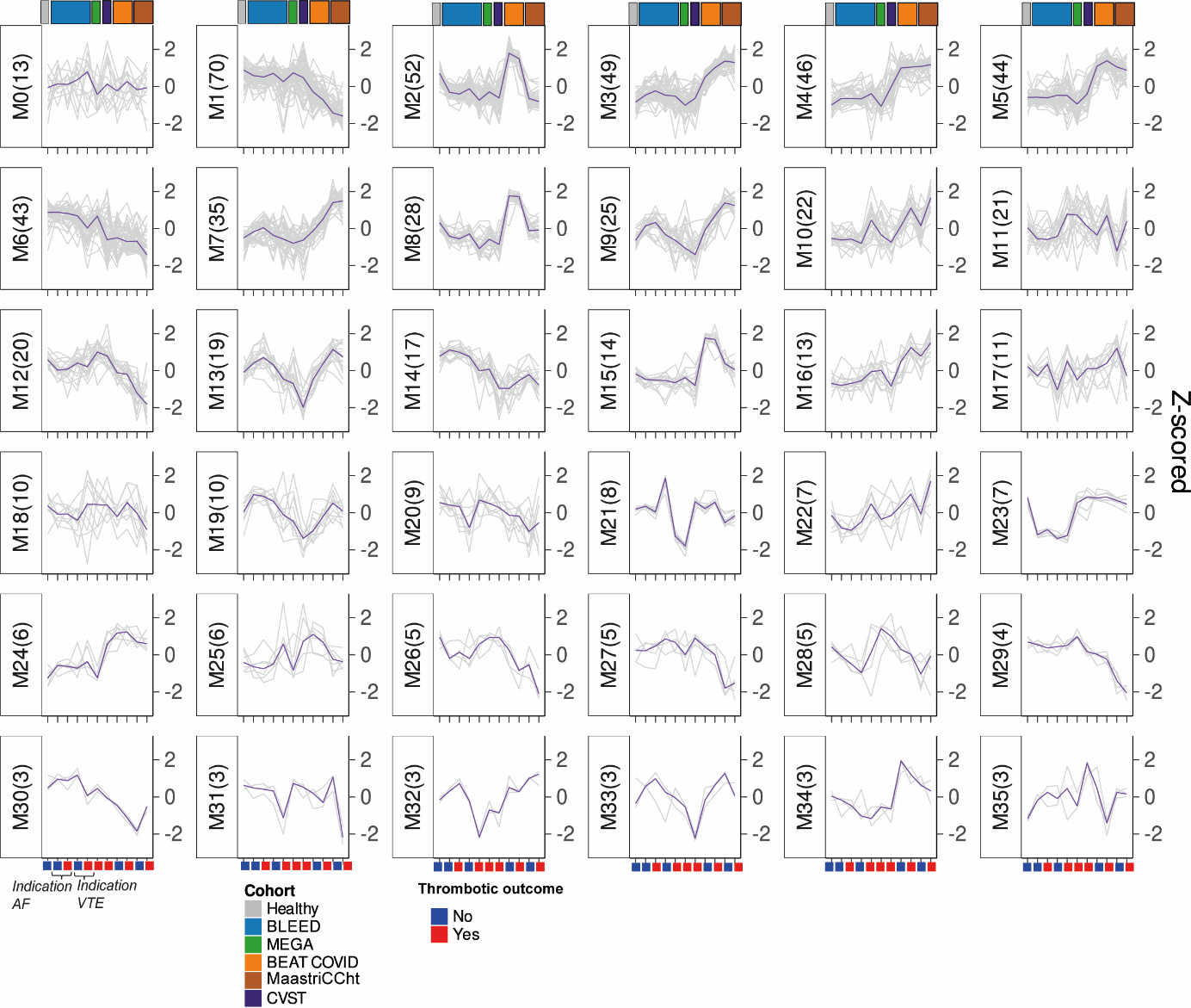


**Supplemental Figure S5:** Line graph depicting Z-scored protein intensities for each cluster. Individual proteins are represented by grey lines, while the median of each cluster is highlighted with a purple line. The graph is stratified across multicentre cohorts based on thrombotic outcomes.
