# Supplementary material for: Exploration of the plasma proteomic profile of patients at risk of thromboembolic events
Source: Res Pract Thromb Haemost. 2025 Feb 28;9(2):102713. doi: 10.1016/j.rpth.2025.102713 (PMC11986537; doi:10.1016/j.rpth.2025.102713)
Supplement: Supplementary Material [file mmc2.docx]

**Supplemental methods:**

**Detailed plasma isolation and collection of laboratory data**

**BLEED study**

From the BLEED study, patients were included from January 2012 until July 2014 from three anticoagulation clinics in the Netherlands [1]. This study included patients at risk of a thromboembolic event, undergoing VKA therapy for atrial fibrillation (AF) or due to a previous VTE. Blood was drawn three weeks after the initiation of VKA therapy. Citrated plasma was obtained by centrifugation of whole blood for 10 minutes at 2800g at room temperature (RT) within 4 hours of sample collection. Plasma samples were stored at -20°C for up to one week, followed by long-term storage at -80°C until further analysis. Patients were routinely monitored following standard procedures of the anticoagulation clinics, including regular measurement of INR. Follow-up started from VKA initiation until a thromboembolic event occurred, VKA treatment ceased, death, the patient moved to an area not covered by the participating anticoagulation clinics or the end of the study (31 December 2014). The BLEED study was approved by the medical ethics committee of the Leiden University Medical Center (LUMC), as previously described.

**MEGA study**

In the MEGA study, patients with a first objectively confirmed deep vein thrombosis or pulmonary embolism were enrolled between 1999 and 2004 from six anticoagulation clinics in the Netherlands [2]. Blood samples were taken at least 3 months after stopping VKA therapy. Citrated plasma samples were obtained by centrifugation whole blood for 10 minutes at 2000*g* at RT and stored at -80°C until further processing. The MEGA study was approved by the medical ethics committee of the LUMC, written informed consent was obtained from all the participants and the study was conducted following the principles of the Declaration of Helsinki.

**CVST study**

Plasma samples from ten individuals with a CVST who were admitted to the Amsterdam University Medical Center (AMC) location Amsterdam, the Netherlands were selected [3]. Patients were diagnosed with a CVST by either magnetic resonance venography or cerebral computer tomography venography as described previously. Citrated plasma samples were collected on admission and stored frozen until laboratory analysis. All participants provided written informed consent. The study received ethical approval from the Canton of Bern in Switzerland and the local AMC Ethical committee in Amsterdam, the Netherlands, and was registered on ClinicalTrials.gov (NCT00924859). Written informed consent was obtained from all participants and the study was conducted following the principles of the Declaration of Helsinki.

**BEAT-COVID study**

From the BEAT-COVID study, 111 plasma samples from 54 individuals who were admitted to the LUMC, Leiden, the Netherlands with PCR-confirmed SARS-CoV-2 infection between April 2020 to January 2021 as previously described [4]. Blood samples were collected shortly after admission and subsequently weekly during the hospital stay. Citrated plasma was obtained by centrifugation of whole blood at 3000*g* for 8 minutes at RT. Clinical data was obtained from the Dutch Covid and Thrombosis Coalition database which included levels of including albumin, urea, hemoglobin, hematocrit, white blood cell count, platelet count, C-reactive protein (CRP), creatinine, bilirubin, aspartate transaminase (AST), alanine transaminase (ALT), fibrinogen, lactate dehydrogenase (LD daily), ferritin, D-dimer, prothrombin time (PT), activated partial thromboplastin time (APTT), and lymphocyte count. All participants provided written informed consent. Ethical approval was obtained from the Medical Ethical Committee Leiden, Den Haag, Delft (NL73740.058.20) and the study was registered in the Dutch Trial Registry (NL8589). Written informed consent was obtained from all participants and the study was conducted following the principles of the Declaration of Helsinki.

**MaastrICCht cohort**

From the MaastrICCht cohort, we selected individuals with PCR-confirmed SARS-CoV-2-infection who were admitted between November 2020 to January 2021 to the Maastricht University Medical Center (MUMC+), Maastricht, the Netherlands [5]. A daily comprehensive and uniform set of clinical and laboratory data (including albumin, urea, hemoglobin, hematocrit, white blood cell count, platelet count, CRP, creatinine, bilirubin, AST, ALT, fibrinogen, LD daily, ferritin, D-dimer, PT, APTT, and lymphocyte count) was collected. Furthermore, left-over citrated blood was collected at least once weekly for storage of platelet-free plasma which were yielded for the current analyses. Citrated platelet-free plasma was obtained using two subsequent centrifugation steps, including an initial centrifugation of 2490*g* for 5 minutes at RT followed by 10.000*g* for 10 minutes at RT. The local institutional review board (Medisch Ethische Toetsingscomissie (METC) 2020-1565/ 300523) of the MUMC+ approved the study. Ethical approval has been obtained from the medical ethics committee (Medisch Ethische Toetsingscommissie 2020-1565/3 00 523) of the Maastricht University Medical Centre+ (Maastricht UMC+), which will be performed based on the Declaration of Helsinki.

**Healthy controls**

From Sanquin, Amsterdam, the Netherlands, we selected plasma samples from 14 healthy donors. Plasma was obtained by centrifugation of citrated whole blood at 120*g* for 20 minutes at RT, followed by centrifugation of platelet-rich plasma at 2000*g* for 10 minutes at RT and platelet-poor plasma at 10000*g* for 10 minutes at RT. Plasma aliquots were stored at -80°C until analysis. Ethical approval was obtained from the Sanquin Ethical Advisory Board.

From the MEGA study, we selected samples from ten healthy individuals that were included in the control group, and therefore did not have VTE. Plasma was obtained by centrifugation as previously described for the MEGA study.

From the POT-KAST study, citrated plasma was obtained by centrifugation of whole blood at 2500*g* for 10 minutes at RT and stored at -80°C within 4 hours after blood collection [6]. Ten healthy individuals who had an indication for a knee-arthroscopy were selected. The protocol was approved by the medical ethics committee at the LUMC.

From the BEAT-COVID study, plasma was obtained by centrifugation of whole blood at 3000*g* for 8 minutes at RT. All participants provided written informed consent and the protocol was approved by the medical ethics committee at LUMC.

**Detailed plasma proteomic workflow**

**Sample preparation**

Plasma samples were thawed at 37°C and 10 μL citrated plasma was transferred to a 96-well plate. Additionally, 10 μL quality control (QC) sample, consisting of pooled plasma from 40 healthy blood donors (Sanquin, the Netherlands) was added to a 96-well plate in triplicate, serving as technical control for the sample preparation, liquid chromatography and mass spectrometry analysis [7]. Plasma was diluted 1:60 with 100 mM tris(hydroxymethyl)aminomethane hydrochloride (Tris, life technologies, UK) (pH=8.0) and 9 μL diluted plasma was mixed with 5 μL of 20 mM Tris(2-carboxyethyl)phosphine (Thermo Fisher Scientific, Rockford, IL)/80 mM chloroacetamide (Sigma Aldrich, St Louis, MO) in 100 mM Tris buffer (pH=8.0). After incubation at 95°C for 5 minutes, samples were cooled down to RT and proteins were digested overnight at 25°C with 200 ng MS-grade trypsin Gold (Promega, Madison, WI) in 50 mM Tris buffer (pH=8.0). Samples were acidified to a final concentration of 1% (v/v) trifluoroacetic acid (Thermo Fisher Scientific, Rockford, IL) and diluted with 0.1% formic acid (Biosolve, the Netherlands) to a final concentration of 500 ng per 20 μL and stored at -20 °C until MS analysis.

**Mass spectrometry analysis**

Each tryptic digest was transferred to an Evotip Pure (Evosep, Denmark) according to the manufactures guidelines and separated on a 15 cm × 150 μm, 1.5 μm Performance Column (EV1137 from EvoSep) with an Evosep One liquid chromatography (LC) system (Evosep, Denmark) on a 30 samples per day (SPD) program [8]. Mobile phase A was composed of 0.1% formic acid and mobile phase B of 0.1 % formic acid in acetonitrile (Biosolve, the Netherland). Peptides were ionized by electrospray and introduced into the Orbitrap Fusion Lumos Tribrid mass spectrometer (Thermo Fisher scientific, San Jose, CA). Data was acquired in data independent acquisition (DIA) mode with a MS1 scan from 390 to 1010 *m/z*, performed at 60K resolution (AGC target of 4 x 10^5^) and a maximum injection time of 100 ms. MS/MS data acquisition was performed in centroid mode. DIA segments/spectra were acquired at 30K resolution (with an AGC target 15 x 10^4^) with 75 MS2 scans using an isolation window of 8 *m/z* and a maximum injection time set on 54. HCD fragmentation was used with a normalized collision energy of 23%. A default charge state of 2 was used. The setting “inject ions for all available parallelizable time” was set.

1 Rein N Van, Lijfering WM, Bos MHA, Herruer MH, Vermaas HW, Meer FJM Van Der, Reitsma PH. Objectives and Design of BLEEDS : A Cohort Study to Identify New Risk Factors and Predictors for Major Bleeding during Treatment with Vitamin K Antagonists. *PLoS One* 2016; **11**: 1–12.

2 Blom JW, Doggen CJM, Osanto S, Rosendaal FR. Malignancies, Prothrombotic Mutations, and the Risk of Venous Thrombosis. *Jama* 2005; **293**: 715–22.

3 Heldner MR, Zuurbier SM, Li B, Von Martial R, Meijers JCM, Zimmermann R, Volbers B, Jung S, El-Koussy M, Fischer U, Kohler HP, Schroeder V, Coutinho JM, Arnold M. Prediction of cerebral venous thrombosis with a new clinical score and D-dimer levels. *Neurology* United States; 2020; **95**: e898–909.

4 Roukens AHE, Pothast CR, König M, Huisman W, Dalebout T, Tak T, Azimi S, Kruize Y, Hagedoorn RS, Zlei M, Staal FJT, de Bie FJ, van Dongen JJM, Arbous SM, Zhang JLH, Verheij M, Prins C, van der Does AM, Hiemstra PS, de Vries JJC, et al. Prolonged activation of nasal immune cell populations and development of tissue-resident SARS-CoV-2-specific CD8+ T cell responses following COVID-19. *Nat Immunol* 2022; **23**: 23–32.

5 Tas J, Van Gassel RJJ, Heines SJH, Mulder MMG, Heijnen NFL, Acampo-De Jong MJ, Bels JLM, Bennis FC, Koelmann M, Groven RVM, Donkers MA, Van Rosmalen F, Hermans BJM, Meex SJR, Mingels A, Bekers O, Savelkoul P, Oude Lashof AML, Wildberger J, Tijssen FH, et al. Serial measurements in COVID-19-induced acute respiratory disease to unravel heterogeneity of the disease course: Design of the Maastricht Intensive Care COVID cohort (MaastrICCht). *BMJ Open* 2020; **10**: 1–8.

6 Raymond A. van Adrichem, M.D., Banne Nemeth, M.D., Ale Algra, M.D., Ph.D., Saskia le Cessie, Ph.D., Frits R. Rosendaal, M.D., Ph.D., Inger B. Schipper, M.D., Ph.D., Rob G.H.H. Nelissen, M.D., Ph.D., and Suzanne C. Cannegieter, M.D., Ph.D., for the POT-KAS for the P-K and P-CG. Thromboprophylaxis after Knee Arthroscopy and Lower-Leg Casting. *N Engl J Med* 2016; : 515–25.

7 Bittremieux W, Tabb DL, Impens F, Staes A, Timmerman E, Martens L, Laukens K. Quality control in mass spectrometry-based proteomics. *Mass Spectrom Rev* United States; 2018; **37**: 697–711.

8 Bache N, Geyer PE, Bekker-Jensen DB, Hoerning O, Falkenby L, Treit P V, Doll S, Paron I, Müller JB, Meier F, Olsen J V, Vorm O, Mann M. A Novel LC System Embeds Analytes in Pre-formed Gradients for Rapid, Ultra-robust Proteomics. *Mol Cell Proteomics* United States; 2018; **17**: 2284–96.
